# Frequency, Severity, Risk Factors, and Outcome of Hemorrhagic Transformation in Anterior and Posterior Stroke

**DOI:** 10.3390/jcm13072010

**Published:** 2024-03-29

**Authors:** Tanya Ayub, Awini Barwari, Josef Finsterer

**Affiliations:** 1 Department of Dermatology, Medical University of Vienna, 1090 Vienna, Austria; 2 Neurology & Neurophysiology Center, 1180 Vienna, Austria

**Keywords:** stroke, hemorrhagic transformation, anterior circulation, posterior circulation, ischemic stroke

## Abstract

**Background**: There are few data on hemorrhagic transformation in posterior circulation strokes (PCS) compared to anterior circulation strokes (ACS). The aim of this study was to retrospectively analyze the incidence of hemorrhagic transformation, its different subtypes, the associations with different risk factors, and the outcome of ACS and PCS patients. **Methods**: A retrospective analysis of consecutive ischemic stroke patients with hemorrhagic transformation was performed. Clinical and demographic data were collected from electronic patient records. **Results**: Included were 186 ACS patients and 67 PCS patients. The median age was 77 years, with PCS patients being slightly younger than ACS patients. ACS patients were more likely to be treated with acetylsalicylic acid before stroke. ACS and PCS patients had comparable frequencies and severity of hemorrhagic transformation. After excluding ACS patients who received thrombectomy, PCS patients developed hemorrhagic transformation more frequently compared to ACS patients. Risk factors for hemorrhagic transformation did not differ between ACS and PCS patients and included vitamin K antagonist use before stroke and thrombectomy in ACS patients. There was no correlation between hemorrhagic transformation and stroke outcome. **Conclusions**: Hemorrhagic transformation occurs with similar frequency in PCS and ACS patients but is more common in PCS patients after the exclusion of ACS patients undergoing thrombectomy.

## 1. Introduction

Lifestyle changes such as decreased physical activity and increased intake of high-caloric food, salt, saturated fats, and trans fatty acids have increased the prevalence of thromboembolic or atherosclerotic events, such as acute ischemic stroke (AIS) and myocardial infarction, in the Western world in recent decades and, more recently, in developing countries [1]. However, due to better preventative measures such as antithrombotic drugs, anticoagulation, statins, antidiabetics, and antihypertensives, stroke incidence and stroke mortality have decreased over the last decades but may be reversing again [2]. Despite these trends, AIS remains one of the five most common causes of death in the US and Europe [3]. Since surviving an AIS often leads to permanent neurological deficits, it is a leading cause of acquired disability in adulthood and is considered the third most common cause after musculoskeletal diseases and mental disorders [4]. 

In general, AIS is categorized as either ischemic, in which the blood supply is interrupted due to obstruction or other mechanisms, or as hemorrhagic, in which a ruptured blood vessel or aneurysm causes bleeding [5]. In AIS, hemorrhagic transformation (HT), defined as spontaneous bleeding in the original infarct area during the recovery period, is quite common and, depending on the severity, can have a negative impact on the prognosis and outcome of AIS. HT is most commonly classified according to the ECASS II criteria [6]. The main risk factors for HT in AIS are thrombolysis or thrombectomy. Those receiving conservative (antiplatelet) therapy for AIS are not at increased risk of HT. In a meta-analysis of 17 studies using 26 prediction models, effective predictors of HT included atrial fibrillation, NIHSS score, serum glucose levels, moderate or severe leucoaraiosis, hyperdense middle cerebral artery (MCA) sign, large infarct volume, and early signs of infarction [7]. In a study of 224 AIS patients who underwent thrombectomy, 86 experienced HT [8]. Compared to patients without HT, patients with HT were older, less likely to be male, had more diabetes mellitus, had a higher baseline NIHSS score, lower Alberta Stroke Program Early Computed Tomographic (ASPECT) scores, and shorter cerebral circulation time [8]. Multivariable logistic regression suggested that cerebral circulation time was independently associated with HT [8]. In 157 patients with juvenile AIS who did not undergo thrombolysis or thrombectomy, the platelet-to-lymphocyte ratio (PLR) was predictive of HT [9]. From a study of 32 patients with AIS who were not treated with thrombolysis or thrombectomy, it was concluded that balanced gut microbiota may prevent HT of AIS [10]. Other established risk factors for HT include white matter lesions (WMLs) and a large infarct volume. 

There are few data on HT in posterior circulation strokes (PCS) compared to anterior circulation strokes (ACS) [11,12]. The aim of this study was thus to retrospectively analyze the incidence of HT, its various subtypes, associations with various risk factors, and stroke outcomes in ACS and PCS patients.

## 2. Material and Methods

### 2.1. Patients

Data from consecutive patients treated for ischemic stroke at the General Hospital Amstetten, Austria, between November 2018 and October 2019, were retrospectively analyzed. All patients diagnosed with acute ischemic stroke during this period were evaluated for inclusion. The diagnosis was made based on the clinical picture and radiologic findings. Imaging was performed using cerebral computed tomography (CCT) or cerebral magnetic resonance imaging (MRI), reviewed by a trained radiologist. 

### 2.2. Inclusion/Exclusion Criteria

This study included all patients with AIS of the anterior or posterior circulation detected by CCT or MRI. Patients with primary hemorrhagic stroke or other neurological diagnoses and traumatic brain injury were excluded from this study. Patients who had both ACS and PCS, which was the case in 13 patients, were also excluded. 

### 2.3. Demographic and Basic Clinical Parameters

Demographic and basic clinical data were gathered from electronic patient records, including age at onset of symptoms, gender, presence of comorbidities, such as diabetes mellitus, arterial hypertension, and dyslipidemia, and any medications the patients were regularly taking before the onset of AIS symptoms. The treatment of AIS with medication, thrombolysis, thrombectomy, or a combination thereof, was also analyzed. 

### 2.4. Imaging

Original imaging data obtained for the diagnosis of AIS were reanalyzed for the presence of microbleeds, infarction size, and the presence of white matter lesions (WMLs), which, if present, were classified as mild/moderate or severe, and whether AIS was located in the anterior or posterior circulation (ACS, PCS). MRIs were also checked for bleeding using susceptibility-weighted imaging (SWI, hemo-sequence (HS)).

### 2.5. Definition of HT

Nowadays, there are two classifications for HT used in the clinical context: the ECASS II classification, which was first introduced for the European Cooperative Acute Stroke Study ECASS II study [6], and the Heidelberg classification [13]. We used the ECASS II classification. The ECASS II classification defines 4 classes of HT: hemorrhagic infarction type 1 (HI-1, petechial hemorrhages at the infarct edges), hemorrhagic infarction type 2 (HI-2, petechial hemorrhages throughout the infarct without mass- effect attributable to it), parenchymal hematoma type 1 (PH-1, ≤30% of the infarct area and minor mass effect due to the hematoma), and parenchymal hematoma type 2 (PH-2, >30% of the infarct area and significant mass effect due to the hematoma [6]. The ECASS II classification is based solely on radiologic findings in CCT scans without considering clinical manifestations. According to this classification, only PH-2 HT is associated with worse outcomes. However, the threshold of >30% of the affected infarct area, which is one of the defining criteria of PH-2, is somewhat arbitrary, and the fact that thrombectomy is now also used in the treatment of AIS has made a new classification (Heidelberg classification) necessary that not only distinguishes between possible symptomatic and asymptomatic intracranial hemorrhages but also includes hemorrhagic events, such as subarachnoid hemorrhages distant from the infarct area and HT caused by thrombectomy. 

### 2.6. Outcome Measures

We chose to use the NIHSS as a parameter for the patient’s clinical presentation and to define whether the patient’s symptoms worsened as a result of HT. All patients were assessed using NIHSS score on the day of admission to the hospital (hospital day 1 (hd1)) and again on hd7 after AIS onset. 

### 2.7. Statistical Analysis

Statistical analysis was performed using Microsoft Excel 2019 and GraphPad Prism 8. With the exception of LDL-C and age (metric), all accumulated variables were dichotomous in nature. Therefore, statistical tests were performed using the chi-square test or Fisher’s exact test. The latter was used if one of the groups had a prevalence of less than five. A simple logistic regression method was performed for metric variables. Nonparametric tests between two groups with ordinal/metric variables were performed using the Mann–Whitney U test. For *p*-values < 0.05, a statistically significant difference between the analyzed groups was assumed. To assess the risk of HT of various independent parameters, odds ratios were calculated and plotted as forest plots. *p*-values < 0.05 were considered statistically significant. Risk factors were analyzed for the entire patient population and separately for patients with ACS or PCS.

## 3. Results

Of the 253 patients included in this study, 186 (73.5%) suffered from ACS, while the remaining 67 patients (26.5%) suffered from PCS (Table 1). The median age of all patients was 77 years, with an interquartile range (IQR) of 66 to 83 years. ACS patients had a median age of 77.5 years, while PCS patients were significantly younger, with a median age of 72 years (*p* = 0.003). AIS was more common in men than women, with approximately 40% female representation in the overall patient population in the ACS (41.9%) and PCS (35.6%) groups. The majority of patients in both the ACS and PCS groups suffered from arterial hypertension, and about one-third of the patients in both groups suffered from diabetes mellitus (Table 1). The average plasma concentration of low-density lipoprotein cholesterol (LDL-C) was also increased. Almost two-thirds of the patients (61.3%) were not taking any medications at the time of stroke onset. This percentage was slightly higher in PCS patients than in ACS patients (Table 1). The most commonly used medication was acetylsalicylic acid (ASA), taken by 28.0% of ACS patients. It was also taken by 14.9% of PCS patients, which was significantly lower (*p* = 0.034) compared to ACS patients. In the entire cohort, 51 patients (20.2%) had microbleeds on admission. The proportion of microbleeds was similar in both groups. WMLs were detected in 176 ACS patients (94.6%). A slightly smaller proportion of PCS patients (*n* = 60, 89.6%) also had WMLs. Treatment was conservative (antiplatelet) in the majority of patients in both groups (Table 1). A significantly higher proportion of ACS patients (*n* = 19, 10.2%) compared to PCS patients (*n* = 1, 1.5%) were treated with a combination of thrombolysis and thrombectomy. Both the NIHSS score at hd1 and on hd7 after admission were significantly higher in ACS patients compared to PCS patients (Table 1).

**Figure 1:**
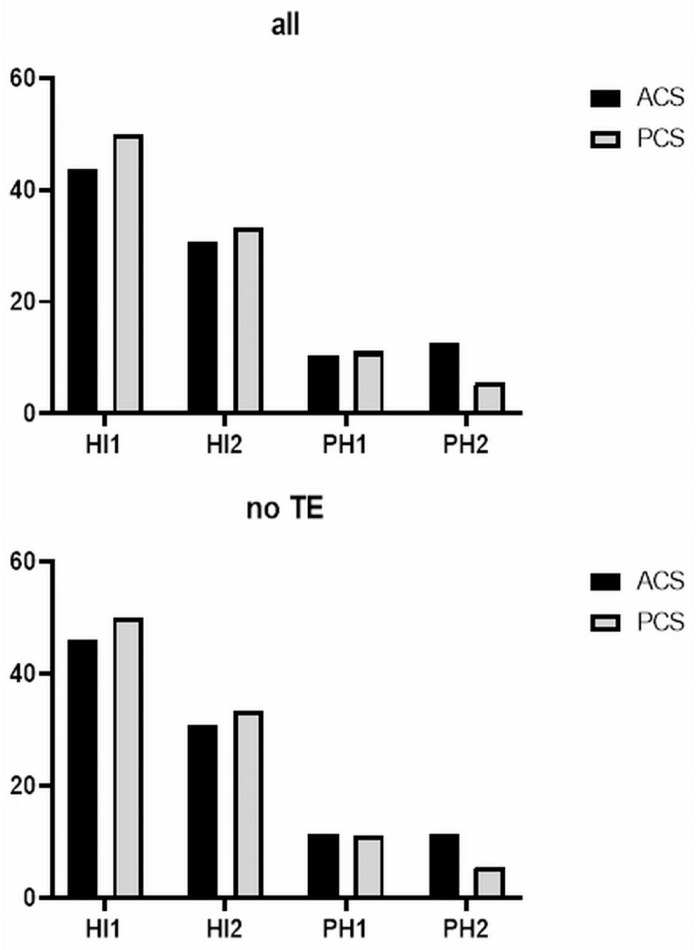
Percentage distribution of ECASS II categories among all HT cases in all ACS and PCS patients (**top**) and in ACS and PCS patients not treated with thrombectomy (**bottom**). HI: hemorrhagic infarction, HT: hemorrhagic transformation, HT: hemorrhagic transformation, PH: parenchymal hematoma, TE: thrombectomy.

In the entire cohort, a total of 57 patients (22.5%) developed HT (Table 2). The incidence was slightly lower in the ACS group than in the PCS group, with *n* = 39 (21.0%) versus *n* = 18 (26.9%), respectively. When comparing the incidence of the specific subtypes of HT according to the ECASS II classification, PCS patients had higher incidences of HI-1, HI-2, and PH-1 compared to ACS patients, but PH-2 was more common in ACS patients (Table 2, Figure 1). The influence of thrombectomy on the occurrence of HT and its severity was also investigated. There was no difference in the incidence of HT or the distribution of the different ECASS II types in all patients depending on whether it was ACS or PCS. No differences were observed between ACS and PCS patients in the overall incidence of HT or patients treated with thrombectomy or conservative (antiplatelet) treatments. However, when considering ACS and PCS patients who did not receive thrombectomy, there was a significantly greater proportion of PCS patients who developed HT compared to ACS patients (*p* = 0.040) (Table 2).

**Figure 2:**
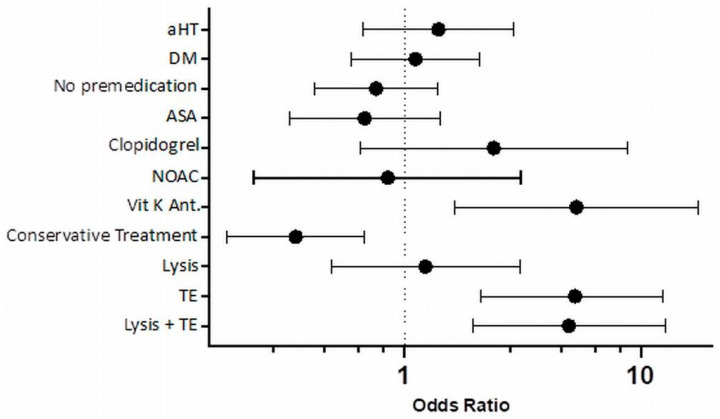
Forest plot depicting the odds ratio (solid black circle) and 95% confidence interval (whisker) of HT risk attributable to various possible risk factors. aHT: arterial hypertension, ASA: acetylsalicylic acid, DM: diabetes mellitus, HT: hemorrhagic transformation, NOACs: new oral anticoagulants, TE: thrombectomy Vit K Ant.: vitamin K antagonists.

Risk factors were analyzed for the entire cohort and separately for patients with ACS or PCS. Arterial hypertension, diabetes mellitus, lack of medication use before stroke, or treatment with new oral anticoagulants (NOACs) before stroke had no influence on the incidence of HT. Likewise, treatment of stroke by thrombolysis had no influence on the risk of HT. The risk of HT was slightly increased in patients who received clopidogrel before stroke onset. Only pre-stroke use of vitamin K antagonists (VKAs) or treatment with thrombectomy alone or thrombolysis plus thrombectomy significantly increased the risk of HT in the entire cohort (Figure 2). Conservative (antiplatelet) treatment and thrombectomy showed opposite associations with the risk of HT, with conservative (antiplatelet) treatment reducing the risk by more than 50%, while thrombectomy with or without concomitant thrombolysis increased the risk of HT by more than four-fold compared to the use of VKAs before stroke. 

All patients were analyzed for NIHSS score on hd1 and again on hd7. When comparing the average improvement by hd7 in patients who developed HT using the NIHSS score and those without HT, there was no significant impact of HT on outcome at this time point. In patients without HT, the NIHSS score on hd7 increased by an average of 1 point, with the 25th percentile showing 2 points and the 75th percentile showing no change. Patients with HT showed no change in NIHSS scores. The 25th percentile represented a 3-point improvement, while the 75th percentile represented no improvement. The comparison between the two groups was not significant. The average improvement in NIHSS scores was −1 in patients from the entire cohort when patients did not develop HT, and it remained unchanged in patients with HT. Similar results were observed when ACS and PCS patients were analyzed separately. A Kruskal–Wallis test revealed that the differences between patients with and without HT were not statistically significant. 

## 4. Discussion

The present study showed that HT occurs with similar frequency in PCS and ACS patients but occurs more frequently in PCS patients after the exclusion of ACS patients undergoing thrombectomy. It was also shown that the severity of HT did not differ significantly between ACS and PCS patients. The presence of arterial hypertension, diabetes, ASA, clopidogrel, NOAC, VKAs, thrombolysis, thrombectomy, and thrombolysis plus thrombectomy did not influence the risk of HT in any of the groups. The stroke outcome did not differ between ACS and PCS patients and HT did not affect the outcome. 

In the present study, HT was slightly more common in PCS patients than in ACS patients. However, the difference was not statistically significant. On the contrary, previous studies found a lower incidence of HT in PCS compared to ACS patients [11,12,14]. It is possible that these differences are due to differences in methodology, for example, differences in diagnostic methods used to detect HT. 

Several risk factors for HT were included in the present analysis but failed to demonstrate a significant effect. One of these risk factors was diabetes mellitus. The data from the literature are contradictory in this regard. In a prospective cohort study of 150 patients with AIS, 55 experienced HT, but diabetes was not found to be a risk factor for HT [15]. In contrast, other studies found that diabetes mellitus is a risk factor for the development of HT [16,17,18]. 

While arterial hypertension is a risk factor for ischemic as well as hemorrhagic strokes, several studies have not confirmed arterial hypertension as a risk factor for HT in AIS [19,20,21,22]. Although it is conceivable that hypertension contributes to the risk of HT, it is possible that the effect is relatively small. Because arterial hypertension is very common in patients with AIS, it is possible that the group of patients without this risk factor was too small to document a significant association between arterial hypertension and HT.

Previous medications, especially taking ASA use or treatment with NOAC, also did not correlate significantly with HT. As with arterial hypertension, it is possible that the number of patients taking these medications was not large enough to detect smaller influences of these factors. This may also apply to previous studies in which no significant effect of antiplatelet therapy before stroke onset or other medications was found [18,23]. This most likely also applies to previous treatment with clopidogrel, which in the present study had an odd ratio of almost 2 for the development of HT. However, this connection did not reach statistical significance either. The risk of clopidogrel was also considered low in a recent study that recommended dual antiplatelet therapy with clopidogrel and ASA to prevent stroke recurrence. This study also included patients with large infarcted areas and those who received thrombolysis or patients with a higher NIHSS score at hospital admission [24]. VKAs are widely known for their association with an increased risk of bleeding events, including hemorrhagic strokes [25,26,27]. However, there are few studies that have investigated this question and clearly indicate that prior VKA use increases the risk of HT in the event of AIS [28]. Consistent with these data, prior use of VKAs increased the risk of HT by more than fourfold, an association that was statistically significant. 

WMLs have been described as risk factors for HT and were more common in ACS than PCS patients but did not reach statistical significance [29,30]. Perhaps due to their high prevalence in both ACS and PCS patients, the group size was not large enough to detect a significant difference. In the present study, both ACS and PCS patients had similar numbers of WMLs. However, consistent with published results, patients with WMLs developed HT more frequently than patients without WMLs in both groups. 

While microbleeds are consistently associated with hemorrhagic strokes or intracranial hemorrhages, the evidence regarding their association with HT is less clear, and they have been reported in some studies as predictive factors for the development of HT in patients with AIS [19,20,23]. In the present study, HT occurred more frequently in patients with microbleeds in both ACS and PCS patients with no differences between groups based on stroke location. In a previous study on 96 patients with PCS, however, lobar topography was an independent risk factor of HT [31]. 

Although it is conceivable that thrombectomy, as an invasive procedure, increases the incidence of HT, there was no difference in the incidence of HT between patients who underwent thrombectomy or not. However, compared to ACS patients, significantly more PSC patients who were not treated with thrombectomy developed HT. The hypothesis that thrombectomy predisposes AIS to HT is still debated in the literature. In recent years, several studies have been published addressing this topic [18,32,33]. In a study on the association of angiographic blush and HT in patients who received thrombectomy, the incidence of HT was 54.2% [33]. This is significantly higher than the incidence of HT in the present study when compared to the entire cohort as well as to ACS patients who received thrombectomy. Other studies also reported higher incidences of HT compared to the present study [17,18,23]. For example, in Salehi Omram’s study [33], this discrepancy was most likely due to the fact that the study cohort included patients with a large infarction who are at increased risk of developing HT compared to the general AIS population. Because the study did not include a control group not treated with thrombectomy, it could not be assessed whether thrombectomy was a significant risk factor for HT. In a study of 289 patients with ACS and atherosclerosis who were treated with thrombectomy plus antiplatelet therapy and carotid stenting, the overall incidence of HT was 35.9% [18]. The higher incidence compared to the present study is most likely due to the presence of additional risk factors to antiplatelet therapy, such as HI-1, HI-2, PH-1, PH-2, intracranial carotid occlusion (both partial and complete), diabetes mellitus, and complete extracranial carotid occlusion with bilateral carotid stenting [18]. The risk of PH-2 after thrombectomy is increased not only in patients with large ischemic core but also in patients who require an increased number of passes during thrombectomy and when thrombectomy is not performed under general anesthesia [34]. In a study of 1400 AIS patients undergoing thrombectomy, high NIHSS, a low ASPECT score, and a long onset-to-groin time were predictors of HT [35]. While some of these studies demonstrated a higher incidence of HT in patients undergoing thrombectomy compared to published overall rates of HT in AIS patients, the lack of comparable control groups does not allow conclusions to be drawn about whether thrombectomy is actually associated with an increased risk of HT. 

The outcome of patients included in the present study did not differ depending on whether the stroke was located in the anterior or posterior circulation or whether the patients experienced HT or not. There are conflicting reports in the literature regarding the outcome of ACS and PCS. While some authors reported that the outcome of PCS is worse than that of ACS, others claimed that ACS and PCS are similar not only with respect to risk factors and etiology but also in comparing long-term outcomes [12,36,37,38,39]. The reason why HT did not influence the outcome in the present study is most likely because only a few patients developed PH-2. Accordingly, it has been previously reported that only PH-type HT can have a negative impact on the long-term outcome of stroke or lead to clinical decline during recovery in the acute stroke phase [13,40]. In some studies, PH-1 and PH-2 were significantly correlated with mortality [18]. The fact that only PH-2 is associated with a significant worsening of long-term outcomes and a higher risk of clinical deterioration, was also found in another study [41]. Patients with PH-2 HT also had the worst 90-day outcome in this study [41]. 

Limitations of this study are that the group sizes were small, that it had a single center and retrospective design, and that stroke was not diagnosed by MRI in all patients. 

## 5. Conclusions

This study shows that HT occurred with the same frequency in ACS and PCS patients. However, excluding ACS patients receiving thrombectomy, a significantly larger proportion of PCS patients developed HT compared to ACS patients. The risk factors for HT were similar in both groups and may be due to the small number of patients with the most severe form of HT (PH-2). This is the only form of HT reported to be associated with clinical deterioration and poor prognosis. No significant association was found between the occurrence of HT and poor outcomes. In summary, the results indicate that further studies with larger groups are needed to evaluate the risk factors of HT in ACS compared to PCS patients.

## Figures and Tables

**Table 1:** Demographic and basic clinical data of the enrolled patients.

Parameter	All (*n* = 253)	ACS (*n* = 186)	PCS (*n* = 67)	*p*-Value
Patient data				
Age, y (IQR)	77 (66,83)	77.5 (68,84)	72 (58,80)	0.003
Females, *n* (%)	102 (40.3)	78 (41.9)	24 (35.8)	0.128
Arterial hypertension, *n* (%)	193 (76.3)	145 (78)	48 (71.6)	0.297
Diabetes mellitus, *n* (%)	84 (33.2)	60 (32.3)	24 (35.8)	0.595
LDL, mmol/L (IQR)	110 (84,139)	111(87,136)	106 (78.5,140.5)	0.729
Previous medications				
No medications. *n* (%)	155 (61.3)	109 (58.6)	46 (68.7)	0.148
ASA. *n* (%)	62 (24.5)	52 (28)	10 (14.9)	0.034
Clopidogrel. *n* (%)	10 (4)	6 (3.2)	4 (6)	0.323
NOAC. *n* (%)	15 (5.9)	12 (6.5)	3 (4.5)	0.765
Dabigatran. *n* (%)	3 (1.2)	3 (1.6)	0 (0)	N/A
Rivaroxaban. *n* (%)	4 (1.6)	3 (1.6)	1 (1.5)	N/A
Apixaban. *n* (%)	4 (1.6)	3 (1.6)	1 (1.5)	N/A
Edoxaban. *n* (%)	4 (1.6)	3 (1.6)	1 (1.5)	N/A
Vitamin K antagonist. *n* (%)	12 (4.7)	8 (4.3)	4 (6)	0.523
Imaging				
MRI, *n* (%)	182 (71.9)	126 (67.7)	56 (83.6)	0.013
MRI with HS, *n* (%)	154 (60.9)	104 (55.9)	50 (74.6)	0.007
Microbleeds, *n* (%)				
<10, *n* (%)	44 (17.4)	32 (17.2)	12 (17.9)	0.853
>10, *n* (%)	7 (2.8)	5 (2.7)	2 (3)	>0.999
WML, *n* (%)				
Mild, *n* (%)	83 (32.8)	62 (33.3)	21 (31.3)	0.184
Moderate–severe, *n* (%)	153 (60.5)	114 (61.3)	39 (58.2)	0.167
Therapy-Modality				
Conservative (antiplatelet) treatment, *n* (%)	202 (79.8)	143 (76.9)	59 (88.1)	0.051
Thrombolysis, *n* (%)	27 (10.7)	21 (11.3)	6 (9)	0.818
Thrombectomy, *n* (%)	4 (1.6)	3 (1.6)	1 (1.5)	>0.999
Thrombolysis and thrombectomy, *n* (%)	20 (7.9)	19 (10.2)	1 (1.5)	0.031
Parameter				
NIHSS at hd1 (IQR)	4 (2,9)	5 (2,10)	2 (1,5)	<0.001
NIHSS at hd7 (IQR)	2 (0.5,7)	3 (1,8)	1 (0,3)	0.006

ASA: acetylsalicylic acid, hd1: hospital day 1, HS: hemo-sequences, IQR: interquartile range, LDL: low-density lipoprotein, N/A: not applicable, NOAC: new oral anticoagulants, WML: white matter lesions, data in parenthesis represent % values.

**Table 2:** HT in ACS and PCS patients.

Parameter	All (*n* = 253)	ACS (*n* = 186)	PCS (*n* = 67)	*p*-Value
HT (total), *n* (%)	57 (22.5)	39 (21.0)	18 (26.9)	0.322
HI type 1, *n* (%)	26 (10.3)	17 (9.1)	9 (13.4)	0.294
HI type 2, *n* (%)	18 (7.1)	12 (6.5)	6 (9.0)	0.439
PH type 1, *n* (%)	6 (2.4)	4 (2.2)	2 (3.0)	0.644
PH type 2 *, *n* (%)	9 (3.6)	8 (4.3)	1 (1.5)	0.691
Treatment type				
Antiplatelet	37 (18.3)	22 (15.4)	15 (25.4)	0.094
i.v. thrombolysis only	7 (25.9)	4 (19.0)	3 (50.0)	0.290
TE only	2 (50.0)	2 (66.7)	0 (0)	N/A
Thrombolysis + TE	11 (55.0)	11 (57.9)	0 (0)	N/A
Antiplatelet + i.v. thrombolysis (without TE)	44 (19.2)	26 (15.9)	18 (27.7)	0.040

*: PH type 2 includes subarachnoidal hemorrhages according to Heidelberg Classification. HI: hemorrhagic infarction, HT: hemorrhagic transformation, N/A: not applicable, PH: parenchymal hematoma, TE: thrombectomy.

## Data Availability

All data presented in the study are available from the corresponding author.

## References

[1] Rethemiotaki I. (2023). Global prevalence of cardiovascular diseases by gender and age during 2010–2019. Arch. Med. Sci. Atheroscler. Dis..

[2] de Havenon A., Zhou L.W., Johnston K.C., Dangayach N.S., Ney J., Yaghi S., Sharma R., Abbasi M., Delic A., Majersik J.J. (2023). Twenty-Year Disparity Trends in United States Stroke Death Rate by Age, Race/Ethnicity, Geography, and Socioeconomic Status. Neurology.

[3] Guzik A., Bushnell C. (2017). Stroke Epidemiology and Risk Factor Management. Continuum.

[4] Adamson J., Beswick A., Ebrahim S. (2004). Is stroke the most common cause of disability?. J. Stroke Cerebrovasc. Dis..

[5] Takashima N., Arima H., Kita Y., Fujii T., Miyamatsu N., Komori M., Sugimoto Y., Nagata S., Miura K., Nozaki K. (2017). Incidence, Management and Short-Term Outcome of Stroke in a General Population of 1.4 Million Japanese- Shiga Stroke Registry. Circ. J..

[6] Hacke W., Kaste M., Fieschi C., von Kummer R., Davalos A., Meier D., Larrue V., Bluhmki E., Davis S., Donnan G. (1998). Randomised double-blind placebo-controlled trial of thrombolytic therapy with intravenous alteplase in acute ischaemic stroke (ECASS II). Lancet.

[7] Zhong K., An X., Kong Y., Chen Z. (2024). Predictive model for the risk of hemorrhagic transformation after rt-PA intravenous thrombolysis in patients with acute ischemic stroke: A systematic review and meta-analysis. Clin. Neurol. Neurosurg..

[8] Liu J., Gu Y., Zhang D.Z. (2024). Cerebral circulation time on DSA after thrombectomy associated with hemorrhagic transformation in acute ischemic stroke. Acta Neurochir..

[9] Wen H., Wang N., Lv M., Yang Y., Liu H. (2023). The early predictive value of platelet-to-lymphocyte ratio to hemorrhagic transformation of young acute ischemic stroke. Asian Biomed..

[10] Huang Q., Wei M., Feng X., Luo Y., Liu Y., Xia J. (2024). Hemorrhagic transformation in patients with large-artery atherosclerotic stroke is associated with the gut microbiota and lipopolysaccharide. Neural Regen. Res..

[11] Demirtas B.S., Ocek L., Zorlu Y., Oztekin O. (2019). Factors Associated with Hemorrhagic Transformation in Infarctions Involving the Posterior Circulation System. J. Stroke Cerebrovasc. Dis..

[12] Pagola J., Ribo M., Alvarez-Sabin J., Rubiera M., Santamarina E., Maisterra O., Delgado-Mederos R., Ortega G., Quintana M., Molina C.A. (2011). Thrombolysis in anterior versus posterior circulation strokes: Timing of recanalization, ischemic tolerance, and other differences. J. Neuroimaging.

[13] von Kummer R., Broderick J.P., Campbell B.C., Demchuk A., Goyal M., Hill M.D., Treurniet K.M., Majoie C.B., Marquering H.A., Mazya M.V. (2015). The Heidelberg Bleeding Classification: Classification of Bleeding Events After Ischemic Stroke and Reperfusion Therapy. Stroke.

[14] Valentino F., Gentile L., Terruso V., Mastrilli S., Aridon P., Ragonese P., Sarno C., Savettieri G., D’Amelio M. (2017). Frequency and determinants for hemorrhagic transformation of posterior cerebral stroke: Posterior ischemic stroke and hemorrhagic transformation. BMC Res. Notes.

[15] Costru-Tasnic E., Gavriliuc M., Manole E. (2023). Serum biomarkers to predict hemorrhagic transformation and ischemic stroke outcomes in a prospective cohort study. J. Med. Life.

[16] Wen L., Zhang S., Wan K., Zhang H., Zhang X. (2020). Risk factors of haemorrhagic transformation for acute ischaemic stroke in Chinese patients receiving intravenous thrombolysis: A meta-analysis. Medicine.

[17] Xu X., Wang D., Wang F., Norton C., Liu X., Selim M. (2018). The Risk of Hemorrhagic Transformation After Thrombolysis for Acute Ischemic Stroke in Chinese Versus North Americans: A Comparative Study. J. Stroke Cerebrovasc. Dis..

[18] Zhu F., Labreuche J., Haussen D.C., Piotin M., Steglich-Arnholm H., Taschner C., Papanagiotou P., Lapergue B., Dorn F., Cognard C. (2019). Hemorrhagic Transformation After Thrombectomy for Tandem Occlusions. Stroke.

[19] Dar N.Z., Ain Q.U., Nazir R., Ahmad A. (2018). Cerebral Microbleeds in an Acute Ischemic Stroke as a Predictor of Hemorrhagic Transformation. Cureus.

[20] Nagaraja N., Tasneem N., Shaban A., Dandapat S., Ahmed U., Policeni B., Olalde H., Shim H., Samaniego E.A., Pieper C. (2018). Cerebral Microbleeds are an Independent Predictor of Hemorrhagic Transformation Following Intravenous Alteplase Administration in Acute Ischemic Stroke. J. Stroke Cerebrovasc. Dis..

[21] Terao T., Mishina M., Takumi I., Komaba Y., Mizunari T., Kobayashi S., Yoshida D., Teramoto A. (2012). Early computed tomography signs as early predictors of hemorrhagic transformation under heparinization in patients with cardiogenic embolism. Geriatr. Gerontol. Int..

[22] Wang R., Zeng J., Wang F., Zhuang X., Chen X., Miao J. (2019). Risk factors of hemorrhagic transformation after intravenous thrombolysis with rt-PA in acute cerebral infarction. QJM.

[23] Liu J., Wang D., Li J., Lin J., Xiong Y., Liu B., Wei C., Wu B., Ma Z., Zhang S. (2017). Cerebral Microbleeds Do Not Predict Hemorrhagic Transformation in Acute Ischemic Stroke Patients with Atrial Fibrillation and/or Rheumatic Heart Disease. Curr. Neurovasc. Res..

[24] Darabont R.O., Stoicescu C., Tiu C. (2019). Therapeutic Challenges in Patients with Noncardioembolic Acute Ischemic Stroke in Need of Double Antiplatelet Therapy for Coronary Artery Disease. Am. J. Ther..

[25] Hakimi R., Garg A. (2016). Imaging of Hemorrhagic Stroke. Continuum.

[26] Lip G.Y. (2011). Stroke in atrial fibrillation: Epidemiology and thromboprophylaxis. J. Thromb. Haemost..

[27] Zoppellaro G., Granziera S., Padayattil Jose S., Denas G., Bracco A., Iliceto S., Pengo V. (2015). Minimizing the risk of hemorrhagic stroke during anticoagulant therapy for atrial fibrillation. Expert. Opin. Drug Saf..

[28] Masjuan J., De Felipe A. (2017). Secondary prevention in non-valvular atrial fibrillation patients: A practical approach with edoxaban. Int. J. Neurosci..

[29] Fierini F., Poggesi A., Pantoni L. (2017). Leukoaraiosis as an outcome predictor in the acute and subacute phases of stroke. Expert. Rev. Neurother..

[30] Nighoghossian N., Abbas F., Cho T.H., Geraldo A.F., Cottaz V., Janecek E., Mechtouff L., Bischoff M., El Khoury C., Schott A.M. (2016). Impact of leukoaraiosis on parenchymal hemorrhage in elderly patients treated with thrombolysis. Neuroradiology.

[31] Ancelet C., Neveü S., Venditti L., Cortese J., Chassin O., Pelissou C., Berthou E.T., Babin M., Nasser G., Benoudiba F. (2023). Pre-treatment risk markers for hemorrhagic transformation in posterior circulation acute ischemic stroke treated with reperfusion therapy. J. Neurol..

[32] Panni P., Gory B., Xie Y., Consoli A., Desilles J.P., Mazighi M., Labreuche J., Piotin M., Turjman F., Eker O.F. (2019). Acute Stroke with Large Ischemic Core Treated by Thrombectomy. Stroke.

[33] Salehi Omran S., Boddu S.R., Gusdon A.M., Kummer B., Baradaran H., Patel P., Díaz I., Navi B.B., Gupta A., Kamel H. (2018). Angiographic Blush after Mechanical Thrombectomy is Associated with Hemorrhagic Transformation of Ischemic Stroke. J. Stroke Cerebrovasc. Dis..

[34] Alexandre A.M., Scarcia L., Brunetti V., Scala I., Kalsoum E., Valente I., Camilli A., De Leoni D., Colò F., Frisullo G. (2023). Predictors of parenchymal hematoma and clinical outcome after mechanical thrombectomy in patients with large ischemic core due to large vessel occlusion: A retrospective multicenter study. J. Neurointerv. Surg..

[35] Cappellari M., Pracucci G., Saia V., Sallustio F., Casetta I., Fainardi E., Capasso F., Nencini P., Vallone S., Bigliardi G. (2023). Predictors for hemorrhagic transformation and cerebral edema in stroke patients with first-pass complete recanalization. Int. J. Stroke.

[36] Alawieh A., Vargas J., Turner R.D., Turk A.S., Chaudry M.I., Lena J., Spiotta A. (2018). Equivalent favorable outcomes possible after thrombectomy for posterior circulation large vessel occlusion compared with the anterior circulation: The MUSC experience. J. Neurointerv. Surg..

[37] De Marchis G.M., Kohler A., Renz N., Arnold M., Mono M.L., Jung S., Fischer U., Karameshev A.I., Brekenfeld C., Gralla J. (2011). Posterior versus anterior circulation strokes: Comparison of clinical, radiological and outcome characteristics. J. Neurol. Neurosurg. Psychiatry.

[38] Dorňák T., Král M., Hazlinger M., Herzig R., Veverka T., Buřval S., Šaňák D., Zapletalová J., Antalíková K., Kaňovský P. (2015). Posterior vs. anterior circulation infarction: Demography, outcomes, and frequency of hemorrhage after thrombolysis. Int. J. Stroke.

[39] Kwon H., Lee D., Lee D.H., Suh D.C., Kwon S.U., Kang D.W., Kim J.S. (2022). Etiology-Related Outcome of Endovascular Therapy in Posterior Circulation Stroke Compared to Anterior Circulation Stroke. J. Stroke.

[40] Pessin M.S., Del Zoppo G.J., Estol C.J. (1990). Thrombolytic agents in the treatment of stroke. Clin. Neuropharmacol..

[41] Berger C., Fiorelli M., Steiner T., Schäbitz W.R., Bozzao L., Bluhmki E., Hacke W., von Kummer R. (2001). Hemorrhagic transformation of ischemic brain tissue: Asymptomatic or symptomatic?. Stroke.

